# Empowering Healthcare Access: Ethnographic Insights into Sustainable Solutions for a Particularly Vulnerable Tribal Group (PVTG) in South India- a Qualitative Research

**DOI:** 10.12688/f1000research.164492.1

**Published:** 2025-06-17

**Authors:** Biju Soman, Sneha D Mallya, Ashwini Kumar, Unnikrishnan B., Harpreet Kaur, Ranjitha S Shetty

**Affiliations:** 1 Department of Community Medicine, Kasturba Medical College, Manipal, Manipal Academy of Higher Education, Manipal, Karnataka, 576104, India; 2 Department of Community Medicine, Kasturba Medical College, Mangalore, Manipal Academy of Higher Education, Manipal, Karnataka, 576104, India; 3 Division of Epidemiology and Communicable Diseases, Indian Council of Medical Research, Department of Health Research, Ministry of Health and Family Welfare, New Delhi, New Delhi, 110029, India; 4 Centre for Indigenous Population, Department of Community Medicine, Kasturba Medical College, Manipal, Manipal Academy of Higher Education, Manipal, Karnataka, 576104, India

**Keywords:** Barriers, Health equity, Health services accessibility, Tribal, Particularly Vulnerable Tribal Population

## Abstract

**Introduction:**

India’s tribal population accounted for 8.6% of the country’s total population. Despite the advancements in healthcare that have occurred in recent decades, which have greatly improved disease prevention and treatment, certain marginalized and browbeaten tribal communities often encounter significant, yet unnoticed barriers when trying to access healthcare services, irrespective of the services and schemes available for them.

**Objective:**

This study aimed to undermine the confronting and promoting factors that influence the utilization of healthcare services by a Particularly Vulnerable Tribal Group (PVTG) in India.

**Methods:**

An ethnographic fieldwork was undertaken to explore the factors contributing to the persistent and alarming decline in healthcare accessibility among a selected PVTG in the Udupi district, located in the coastal region of southern India. The study involved conducting eight in-depth interviews (IDIs) and three focus group discussions (FGDs) with representatives from the PVTG community, healthcare providers, and key informants associated with institutions delivering healthcare services to this marginalized group.

**Results:**

Numerous barriers have been identified as significant determinants impeding the utilization of healthcare services and schemes by selected PVTG. These barriers encompass the lack of culturally sensitive care, discrimination faced by the community within healthcare establishments, centralized approach to service delivery, and limited collective capacity to advocate for services and schemes that are more inclusive and sustainable for their communities.

**Conclusions:**

Although several influential factors are behind the resistance to utilizing healthcare services and schemes by the PVTG in Udupi district, culturally oriented care, absence of discrimination, decentralized service delivery, and their capacity for collective bargaining might enhance their utilization of healthcare services, and this would enable the improvement of the tribe’s overall well-being and health status.

## Introduction

Tribal health in India is a crucial aspect of the country’s public health landscape as it involves addressing the unique healthcare challenges faced by indigenous communities or tribes residing in various regions. ^1^ Despite advancements in healthcare and medical technologies in the country, accessibility and availability of health services remain meagre among the tribal population because of their geographically isolated habitats. ^2^ The initiatives of the Government of India (GoI) aim to bridge the gap between healthcare services and their accessibility by tribes. A few such measures are the National Health Mission (NHM) and the Tribal Health Research Office (THRO), which encompass targeted strategies and programs to improve healthcare services in tribal areas, based on evidence. This has resulted in the establishment of many health sub-centers and mobile medical units in the tribal areas, yet does not cover all remote areas. ^2, 3^ Efforts to address tribal health in India require a comprehensive approach that combines modern healthcare practices with respect to cultural diversity and the integration of traditional healing methods. ^4^ Tribal healthcare usually falls within the ambit of rural healthcare, as most of the tribes reside in rural areas, they are diverse concerning the terrains in which they live and their socio-cultural systems that make their health needs unique. ^5– 9^

Of the 705 Scheduled Tribes (ST) in India, 75 are categorized as Particularly Vulnerable Tribal Groups (PVTGs); hence, they follow a pre-agricultural system of living and hunting, featuring declining population growth and extremely low levels of literacy, compared to other tribal populations. ^2^ In Karnataka, a southern state in India, there are two PVTGs, Jenukuruba and Koraga, widely spread across the coastal districts of Dakshina Kannada and Udupi. There are 2575 families of the latter, constituting merely 8671 individuals. The problems and health issues of this vulnerable and highly marginalized community are often overlooked. The majority of them are forest dwellers and do not mingle well with mainstream society because of various factors such as distance, language barriers, alcoholism, casteism, social discrimination, and a certain level of stigmatization. ^10, 11^ They also deprive themselves of proper utilization of and access to healthcare services, although there are numerous health problems. Very few studies have been conducted on the healthcare delivery system among tribal groups, especially on PVTGs; however, numerous studies have focused on examining the health beliefs and practices within this community. ^12^ Hence, it is crucial to unearth the factors that hinder the adoption of positive healthcare practices by them. We conducted a qualitative study among this tribal community and concerned stakeholders to evaluate their understanding and perspectives regarding the available public healthcare services and their utilization by this PVTG. The results of this study would help stakeholders and policy makers implement effective strategies to improve the health status of the tribal population, especially when they are categorized as PVTG and their strength is alarmingly declining.

### Purpose of the study

After conducting a sufficient review of the literature and preliminary studies with the selected PVTG, we aimed to explore their health-seeking behavior and identify prevailing barriers and facilitating factors that contribute to the utilization of health care services. The qualitative research tradition adopted in this study is Ethnography. ^13^

### Research framework ( Figure 1)

**Figure 1.**
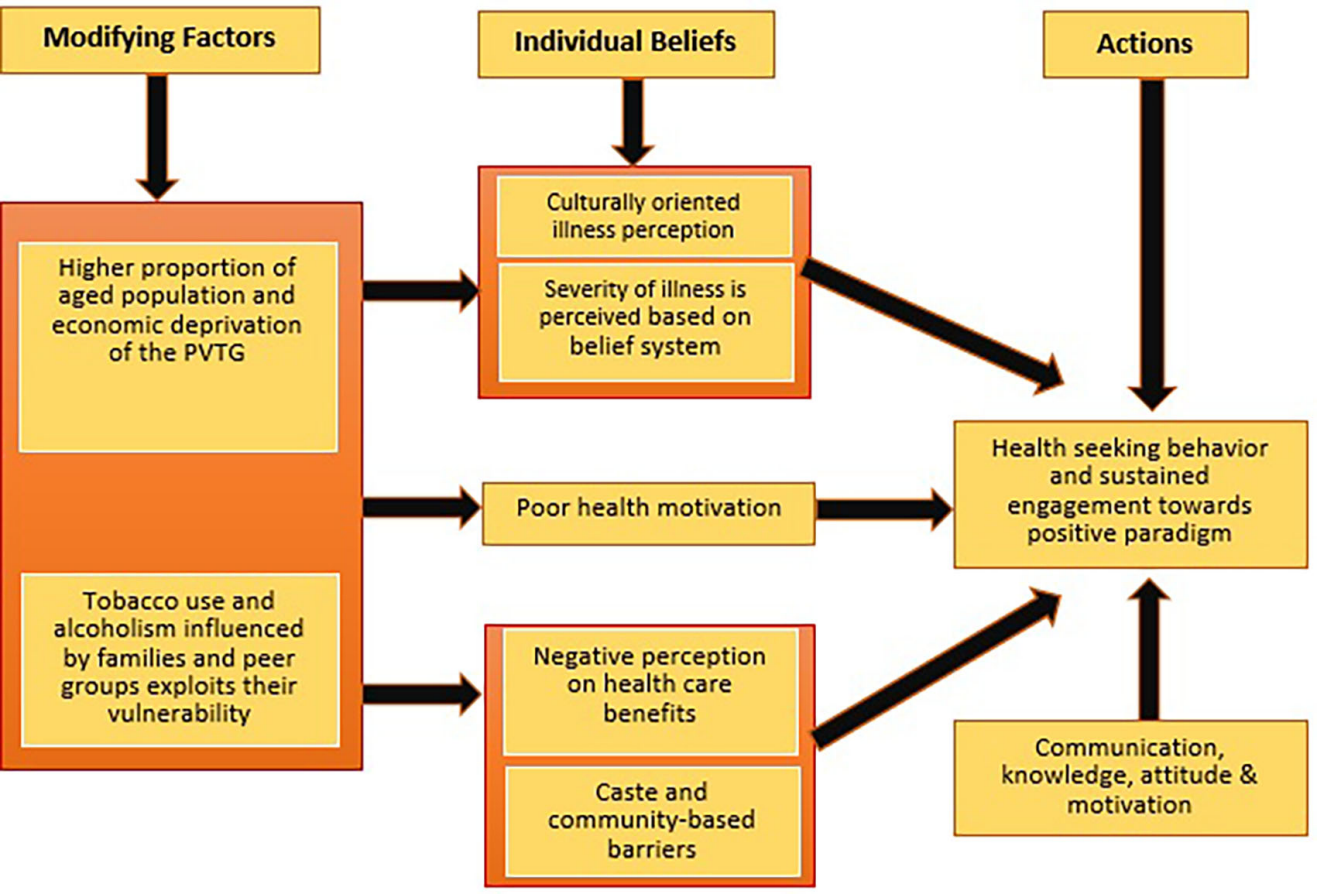
Health Belief Model (HBM) in the context of a PVTG in Udupi district. The HBM recommends enhancing communication, motivation, and engagement to promote a more positive health paradigm.

The framework adopted for this study was the (HBM). ^14^ We gathered information on health beliefs, practices, and factors influencing healthcare to generate scientific evidence for our research question, focusing on the key factors influencing the health-seeking behavior of this tribal population, including their sociocultural beliefs, accessibility to healthcare facilities, and indigenous healthcare practices. ^15^ The majority of this tribal community often adheres to traditional and home remedies, and the prevailing cultural beliefs and social barriers further impede their health-seeking behavior.

## Methodology

### Research design

This ethnographic research was conducted within a PVTG community located across multiple settlements in the Udupi district, alongside the stakeholders engaged in delivering services to this population. Ethnographic methods are particularly effective for understanding interactive dynamics, as they provide both insider (emic) and outsider (etic) viewpoints, enabling objective and thorough analysis and interpretation. Furthermore, ethnography is an important tool for exploring health beliefs and experiences with a specific focus on issues or situations that emerge within cultural or subcultural frameworks.

### Study setting and sampling

This study was conducted using various taluks in the catchment area. The study population consisted of adult individuals from the selected PVTG, Administrative Medical Officers (AMO), healthcare workers, officials from Non-Governmental Organizations (NGO), and the Integrated Tribal Development Project (ITDP), Udupi district. The participants were chosen through a combination of mixed purposeful sampling to facilitate triangulation and flexibility in meeting the needs of multiple stakeholders. This included snowball sampling, homogenous sampling, opportunistic sampling, and intensity sampling, ^16^ in which information-rich participants were identified through a sample frame of key respondents from each category in the inclusion criteria of Focus Group Discussions (FGD) and In-Depth Interviews (IDI). The tribal population who showed resistance to utilization of current health services and those who had experience of health care seeking or followed traditional healing practices were included in the study. ^17^

**Role of the research team**

The research team comprised experts in community medicine (RSS; UB; SDM; AK), who are MD in Community Medicine and are teaching faculty in medical colleges, and (HK) a public health expert and scientist, who were the key people in the methodological approach and design of the study. Further, the research team had a male research scholar (BS) and a male research assistant, both of whom had over ten years of experience in designing and conducting qualitative studies on field activities and data collection processes. RSS is the Principal Investigator (PI) of this project and has authored papers using qualitative research methodologies. BS performed the IDIs and FGDs and the research assistant took part in ice breaking of the sessions, transcription, and translation of the data.

**Ethical considerations**

Permission was obtained from the State Health and Family Welfare Department, Bengaluru, Karnataka, and the ITDP, Udupi, to conduct the study among the selected PVTG. Approval was sanctioned by the Kasturba Medical College and Kasturba Hospital Institutional Ethics Committee (KMC-KH IEC), Manipal Academy of Higher Education, Manipal, Karnataka (IEC No: 778/2019 Approved on 19 November 2019). The participants were explained about purpose of the study by using a Participant Information Sheet (PIS), and Informed Consent (IC) was obtained from each participant before enrolling into the research. The research was conducted as per the Declaration of Helsinki. ^18^ This study was registered on 16 December 2019, under Clinical Trials Registry- India (CTRI/2019/12/022385).

### Data Collection

Ethnographic fieldwork was conducted from April 2022 to March 2023 in the Udupi district of southern Karnataka, India, which included FGDs and IDIs ^11^ among the target groups.

(a) ***FGDs***- One FGD was conducted with the community leaders (key informants) of the PVTG to investigate the health beliefs and practices of their community members. Two FGDs were conducted with the nurses and Accredited Social Health Activists (ASHAs) from selected government healthcare facilities catering to this tribal community. The recruited participants were provided information regarding the date, time, and location of the FGD. Written informed consent was obtained from each participant before recruiting them, and additionally for audio recording of the FGD/IDI after explaining the purpose of the research, using the participant information sheet. Three FGDs were conducted at two selected Primary Health Centres (PHCs) and one community hall located in a tribal colony. There were eight participants in each FGD, which lasted approximately 45 minutes to an hour.
(b) ***IDIs*** –The IDIs were conducted with the key informants from the PVTG community, community health centers (CHCs), PHCs, the ITDP office, a local NGO, and AMOs from PHCs and CHCs of those areas residing in the tribal community. All IDIs took place in respondents’ offices. Before the interviews, participants were briefed on the purpose of the study and detailed the participant information. Each IDI session lasted for approximately 30-45 minutes.

During FGDs and IDIs, the research assistant facilitated the sessions by taking field notes, recording conversations, and capturing non-verbal cues through field notes. ^19^ All sessions were conducted using the FGD schedule and IDI guide, which were prepared, validated, and pilot tested by the research team. Only the consenting respondents were present at the venue, and no other individuals attended the sessions. After consenting, none of the participants refused or dropped out during the sessions. The study was conducted among different groups of respondents in each FGDs and IDIs until data saturation was reached. There were no repeated sessions in the study with the same group or individual respondents. The data and participant identities were treated with complete confidentiality, and the recordings and transcripts were stored securely.

**Reflexivity and rigor**

To establish a connection with the interviewees and key stakeholders, the research team attended various meetings conducted periodically at the NGO office and district administrative office, along with leaders of the selected PVTG community, to ensure that bias and assumptions were eliminated in the study process. In situations where the research team faced difficulty in accessing the tribal hamlets and establishing rapport due to language and distance barriers, assistance was sought from the hamlet chief to ensure a smooth communication process. ^20– 22^ We ensured the trustworthiness of the information provided by the participants by adopting multiple data collection methods from stakeholders and beneficiaries of the healthcare services. Thorough feedback from the participants after the collection of data was made by the research team to adhere to the confirmability and credibility of the research process.

**Validity and reliability**

To ensure that the captured data contained the lived reality of the tribal community in accessing healthcare services, rigorous and multiple strategies were adopted, such as various forms of member checking, triangulation, dependability, peer debriefing, and confirmability ^23^ ( Table 1).

**Table 1.** Establishment of rigor based on Lincoln and Guba (1994). ^27^

Criteria	Strategies Employed
Credibility	The selected PVTG community was engaged in extensive fieldwork of our qualitative study, allowing for the collection of data through the active participation of its members, who shared their personal experiences and opinions. To ensure the research's credibility, triangulation and member-checking techniques were employed. The participants were given their transcripts to ensure their content's precision.
Transferability	We have made sure the methodology adheres to scientific and systematic steps of qualitative research; hence the study could be done among other tribal groups elsewhere. Detailed descriptions and interpretations were given for the observations made by the researchers.
Dependability	To facilitate data analysis, the study employed intensity sampling to ensure a comprehensive and detailed description. The participants were thoroughly briefed on the research objective, and their confidentiality was ensured. To prevent any loss of information, a meticulous verbatim transcription was conducted.
Confirmability	To ensure the confirmability of our study, we have diligently upheld the accuracy of participant records and interview details, while adhering to a systematic approach in making theoretical and methodological choices throughout the study. Our commitment to meticulous data recording and management has further bolstered the reliability of our findings.

Rigor of the study was established based on the parameters of Lincoln & Guba.

**Data analysis**

All audio recordings were transcribed and translated verbatim into *Kannada* (native language of Karnataka State, India). To ensure accuracy, the transcriptions were translated into English and cross-checked with the original audio recordings by a language expert who was part of the research team and cross-verified by the principal investigator.

The data analysis process began with each member of the research team individually and repeatedly reading through the transcripts, confirming that the data were rich and sufficient for analysis. The translated scripts were manually coded by two independent reviewers adopting reflective and inductive approaches, and various themes were derived from the coding process, which were then reported and discussed with selected respondents to ensure consistency of the data. ^24^ In cases where there were discrepancies in coding, the team resolved them through negotiated consensus and discussion. ^25^ An unconstrained coding matrix was established, and sub-categories were created under generic categories. ^26^ Transcript coding was performed manually, and the data were organized using an Excel worksheet.

## Results

As part of this study, eight IDIs and three FGDs were conducted among various stakeholders from health and tribal communities ( Table 2).

**Table 2.** Characteristics of the study participants.

Characteristic	AMO	ITDP officials	NGO members	Staff Nurses	ASHA workers	Tribal Key Informants	Total
	*n* = 4	*n* = 2	*n* = 2	*n* = 8	*n* = 8	*n* = 8	n (%)
Sex							
Female	3	1	1	8	8	4	25 (78.0)
Male	1	1	1	0	0	4	7 (22.0)
Age group							
20-35 years	2	0	0	3	6	2	13 (41.0)
36-50 years	2	2	1	4	2	4	15 (46.0)
>50 years	0	0	1	1	0	2	4 (13.0)
Education							
Primary	0	0	0	0	0	3	3 (9.0)
Secondary	0	0	0	0	3	3	6 (19.0)
PUC	0	0	0	0	5	2	7 (22.0)
Graduate	0	2	2	0	0	0	4 (13.0)
Professional Qualification	4	0	0	8	0	0	12 (37.0)

The study participants in each FGD and IDI were homogenous to their own group, which facilitated more focused discussion.

A summary of the general categories and subcategories derived from the qualitative analysis is presented in Table 3.

**Table 3.** Categories identified as barriers and facilitators in the utilization of healthcare services.

Generic Category	Barriers in the utilization of healthcare services		Facilitators in the utilization of healthcare services	
	*Perceived by service providers*	*Perceived by the PVTG*	*Perceived by service providers*	*Perceived by the PVTG*
Predefined bounds/coding rules	Cultural practices and beliefs fortified by lack of awareness and non- participation in the public healthcare programs which interfere with utilization of healthcare services. (AMOs 2, 3 & 4/IDIs)	Attitude of healthcare workers and lack of follow-up in services offered by healthcare facilities caused a hindrance in the utilization of healthcare services. (PVTG Key informants 3, 4, 5 & 6/ (FGD)	Coordinated activities and services by Kasturba Hospital, ITDP and NGO are the greatest facilitating factors to improve the health status of the selected PVTG. (ITDP officials 1 & 2/IDI)	Health education, empowerment of youth, series of healthcare initiatives and involvement of gate keepers in activity implementation are effective facilitators. (PVTG Key informants 3, 4, 5 & 6/ (FGD)
Sub-categories	• *Belief system* • *Traditional practices* • *Lack of knowledge* • *Lack of motivation* • *Non-acceptance of services*	• *Cast and creed* • *Untouchability* • *Reluctance in listening to health problems* • *Negative attitude* • *Inadequate home visits*	• *Planning* • *Coordination* • *Information Education and Communication (IEC) activities* • *Program implementation* • *Program evaluation* • *Sustainability*	• *Coordinated activities* • *Multi-sectoral approach* • *Funding* • *Availability of schemes* • *Self-efficacy *

Barriers and facilitators perceived by the stakeholders and beneficiaries in the healthcare system for the tribal population.

On analyzing the data, the following themes emerged as barriers and facilitating factors that influenced the acceptance and utilization of healthcare services by the tribal community. Even though there are a limited number of facilitating factors, they are not adequately percolated to this tribal community because of the strong influence of some of the barriers, such as lack of awareness, lack of education, unfavorable attitudes, and distance to healthcare facilities. These themes evolved from the categories and subcategories identified in the above table, as expressed by the participants.

### A. Barriers to healthcare utilization

*Traditional practices and culture*

The public health system has evolved through various stages and phases, mainly based on the principles and systems of healing practices that exist in many parts of the world. Modern medicine is based on various models. ^27^ However, the selected PVTG communities still preserve and follow traditional healing practices, especially among the elderly and those living away from mainstream society. Although healthcare services and systems try to percolate into their community, the beliefs and traditional practices of the people are very strong. Some stakeholders have highlighted these points.

*“The health and hygiene practices of the general population are not followed by this tribal group. For example, you have to boil and cool water before you drink, such kinds of things are not practiced by them. During the days when we started working for them, whenever the tribal volunteers got vomiting or diarrhoea, they would not drink water and manage their dehydration, which led to mortality of many of them.”* (AMO/R3, IDI-3)

*“People in our community use home remedies to treat minor ailments such as cold, cough and stomach pain. If the disease is not cured, only then we will go to hospital, for example, in our culture there is a practice of heating the stomach ache by placing a lit bowl that contains mixture of granite and lime. It is our belief that the lamp in the bowl sucks all the pain. We have so much faith in the god. We offer blood of animals or birds to the god, just like our ancestors did. This ritual cures our illness.”* (PVTG Key informant/R1, FGD 1)

*Lack of awareness and non-participation*

Many participants mentioned that the tribal community members do not possess knowledge of schemes and other healthcare facilities offered by the government. Although several coordinated activities are initiated by district administration and NGO, their participation in such programs is suboptimal. ASHAs, ITDP officers, and NGO members had to frequently visit the community at certain intervals to motivate them to feel the need for empowerment. The following discussion of the respondents highlighted the necessity of improving their utilization of healthcare services.

*“Illiteracy is one of the major barriers. They do not understand what facilities are provided to them. During camps for the under privileged communities, we would go to them early morning, and it’s for their benefit, which otherwise they do not avail. For instance, we reach their home before 7.30 am, so that we can take blood samples and treat them before they leave their homes for any work.”* (AMO/R8, IDI-1)

*“During COVID- 19 time, they did not want to accept that the disease is caused by a virus. When they saw the people, who took treatment were getting better, the attitude of these people towards acceptance of medicine was better. The same thing happened with vaccination as well.”* (AMO/R5, IDI-2), respectively.

*“When we intimate pregnant women about hygiene and health of child, they agree to follow that, and later they fail to adhere to that. As the space in their house is too small, it is difficult to take care of the child well, they cook, bathe, and live in the same place. If we tell them to clean the clothes, they will do as their preference.*” (ASHA/R1, FGD 2)

*Mental health and substance abuse*

Mental health issues, as well as alcoholic malnutrition, were also commonly identified. The majority of these patients experience weight loss and anemia. The overall health and nutritional status of mothers and children is unsatisfactory. Poor eating patterns, lack of awareness about methods of cooking, and consumption of nutritious diets further worsen their nutritional status.

*“Illiteracy and alcoholism are the two hindering factors regressing their health status, they should stop consuming alcohol, and learn healthy eating pattern, so that they can improve their health.”* (AMO/R8, IDI 2)

*“When I ask, they do not reveal the details, however very high levels of tobacco and alcohol addiction is observed among them.”* (Staff Nurse/R5, FGD 3)

*“This tribe is eligible for reimbursement of medical expenditure; many people do not take the benefit of this also. They usually have, alcohol addiction and associated medical conditions. We have medical reimbursement for their treatment, if they have to spend for their treatment. However, they won’t go for treatment at all.”* (ITDP official/R2, IDI-2)

“ *We should live in harmony at home, read good books, should avoid bad habits, there should be proper communication with others in the family and neighbourhood, our perception should be good. However, none of these practices exist in the people of our community.*” (PVTG Key informant/R1, FGD- 1)

*“If we try to prove the mental health of the family is good, we need to understand their problems and often need to give counselling to them. However, the key problem here what we face is, the family members, especially women do not share their mental health issues with us.”* (AMO/R7, IDI-7)

*“It is not the stigma or misconception that prevent them from accepting mental health services, but their unfavourable attitude and ignorance prevent them and ultimately they do not accept the services.”* (AMO/R5, IDI- 5)

*Attitude of healthcare workers*

A positive attitude of healthcare providers towards the tribal community can bring about desired changes among them. Tribal community members often report that the attitude of healthcare providers does not always motivate them to approach and accept the services offered by healthcare facilities. The tribal participants emphasized that staff from healthcare facilities, especially those from the upper cast, always demonstrate an unwelcoming attitude and a kind of distancing behavior.

*“According to my experience, once my baby got sick and did not heal in six months, we took the baby to a government hospital. The healthcare providers had a very remarkable way of approach and treated us indifferently, therefore we did not take the medication they gave to us. Despite going to the same hospital for four to six times, the doctor demonstrated unwelcoming attitude and approach and did not talk about the next level of treatment for the baby. I know this was because we belong to a particular tribal group.”* (PVTG Key informant/R6, FGD 1)

The tribal community felt that healthcare personnel and the systems were not considerate towards them; on the contrary, the healthcare personnel expressed disapproval of the tribal group when services were offered to them.

*“After I joined, I had to go to three houses in interior place, which is far away. They were too interior in the forest. It was not possible to go there. And after taking all the difficulty, when I reached there, the people were not cooperative. It was difficult to go everywhere in that situation.”* (ASHA/R7, FGD 2)

*Non-acceptance of services by the PVTG*

Despite several government schemes being provided to them, many at times they do not accept them due to a lack of awareness, which further hinders their progress. While discussing the attitude of this tribal community towards the utilization of healthcare services, the respondents highlighted that they still have culturally based practices that make them reluctant to accept services provided by the government.

*“There are families in the rural areas who are still utilizing only nutritious food provided from ITDP, even though there are a lot of schemes available for us. They do not even know what facilities are available to them and don’t know whom to meet and what to enquire. This is because they do not attend any important meetings held for us, and they do not get full benefit of any project.”* (PVTG Key informant/R4, FGD- 1)

*“Actually, minor illnesses would be treated at home initially most of the time, and later they will be visiting the health care facilities. Even during delivery, they will be having their own dais or elderly woman at their help in the institution too, which I have observed as a best practice among them*”. (AMO/R7, IDI-7)

*“Recently we could help out one primigravida lady, who was reluctant to move to PHC for her delivery. Ambulance could not reach that place as early as we expected due to some unavoidable reasons. However, after our healthcare team could access her, they assisted her in further needs and brought her to a safer delivery at the PHC.”* (AMO/R8, IDI-8)

*“We have scarcity of data on their health issues, we need to maintain and access the data regarding it. Then only we can provide them healthcare services based on their needs”* (ITDP official/R1, IDI 1)

### B. Facilitators for utilization of healthcare services

*Implementation and sustenance of community-based programs in tribal regions*

There are a lot of programs initiated by the government and NGO, and various social welfare agencies for this tribe. However, the sustainability of the programs depends on funding, manpower, availability of other resources and infrastructure, and the successful implementation of the programs. Hence, a strong policy-based plan that focuses on the improvement of tribal groups must be established to attain long-term goals.

*“When we conduct health camps in the tribal hamlets, majority of them will not participate in that. In such cases we need to conduct the camps at least for four days continuously. It needs a lot of efforts, IEC strategies and coordination. If the tribal members do not make use of it, these programs are failures.”* (ITDP official/R2, IDI-2)

*“Government is initiating a lot of programs for us, but many at times, we do not follow-it up and loose the sustainability of it.”* (Key informant/R2, FGD- 1)

Although many programs have been initiated for this PVTG by various agencies and NGOs in the past years, the objectives and outcomes of the programs, especially among vulnerable populations, are successful only if such programs are sustainable. Respondents reiterated this point, as mentioned below.

*“Whatever the government programs are initiated, first preference is given for this particular tribal community. ITDP, municipal council, or there may be the tribal health department, whichever may be the government agency concerned with any scheduled tribes, 25% of the total amount of financial grants will be spent for the under-privileged target group.”* (ITDP official/R4, IDI-4)

*“Our NGO was established in 1987. We receive funds from various charitable organizations. In 1987, we started working with this PVTG community on a small scale, in 1990s, we worked in about 2-3 taluks. In the year 2002-2003 we extended our work to more districts and after 2003, we got acknowledgement for our welfare work among this tribe.”* (NGO Coordinator/R3, IDI-3)

*Inter-sectoral cooperation in community enhancement*

Inter-sectoral cooperation is a key element in the success of community-based programs, especially healthcare services. Improvement of the PVTG, especially in health-related matters, is achievable through a collaborative approach from the government, private sectors, and NGO. Some participants shared their experiences and views on the successful operation of the intersectoral collaborative approach in their upliftment.

*“In this PVTG community, one developmental model was evolved while working with them. In this model, the participation of people is a very important element, so in that aspect, all the programs are held by participation of people, may it be planning, identification, or implementation process. Effective participation of people is happening in all stages, because of this model itself. Not only for this community, but this is also a working model for all the tribal communities.”* (NGO Coordinator/R3, IDI-3)

*“We have integrative approach for this tribal community through a private psychiatric hospital in Udupi, and Kasturba Medical College, Manipal to stop alcoholism among them and improve their health status. We cannot do activities without the cooperation of the village panchayats and non -governmental organizations. NGO are doing good job in this regard. The role of these organizations is inevitable for the success of government-initiated programs”.* (ITDP official/R2, IDI- 2)

*“Every household gets food and other grocery items from ITDP, and also we get free treatment provision from private medical college and some selected hospitals, but many of our people do not use these facilities.”* (Key informant/R6, FGD- 1)

**Figure 2.**
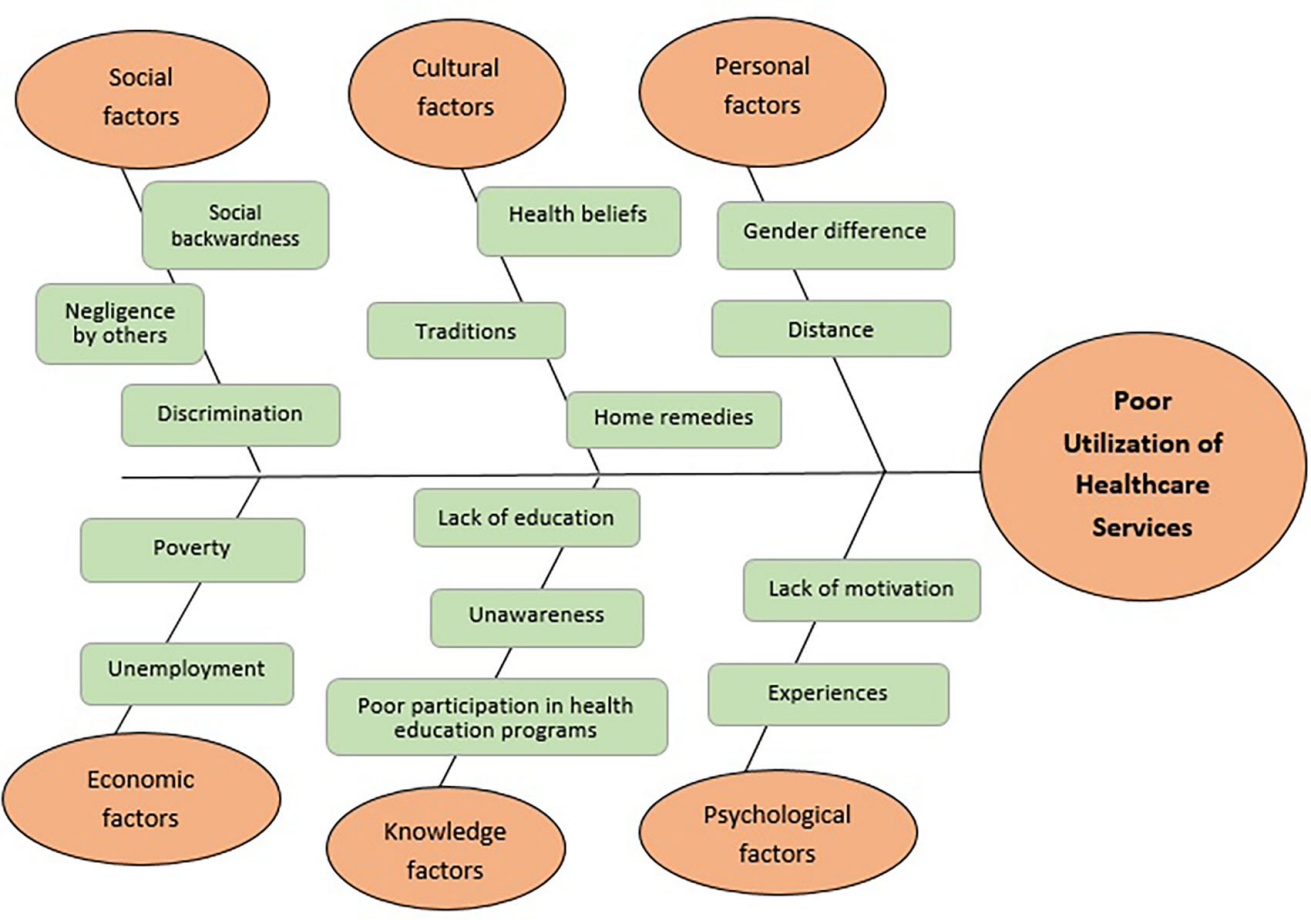
Fishbone diagram representing the cause effect phenomena in the poor utilization of healthcare services by the selected PVTG in Udupi district. The fishbone diagram depicts the causes of poor healthcare utilization among the tribe based on the HBM framework used in this study. This diagram highlights minor themes that contribute to barriers that lead to inadequacy in the utilization of healthcare services by the tribal group under the study. Overcoming these barriers would enable the implementation of facilitating factors more successfully and sustainably to enhance the health status of the tribal community.

The major barriers to the utilization of healthcare services identified from the qualitative analysis were traditional practices and culture, lack of awareness and initiation, alcoholism and malnutrition, unfavorable attitude of healthcare providers, and non-acceptance of services by this PVTG community, which has been depicted in the fish-bone diagram as a cause–effect relationship ( Figure 2).

**Inductive approach to suggest improvement of health status of tribal population**

**Figure 3.**
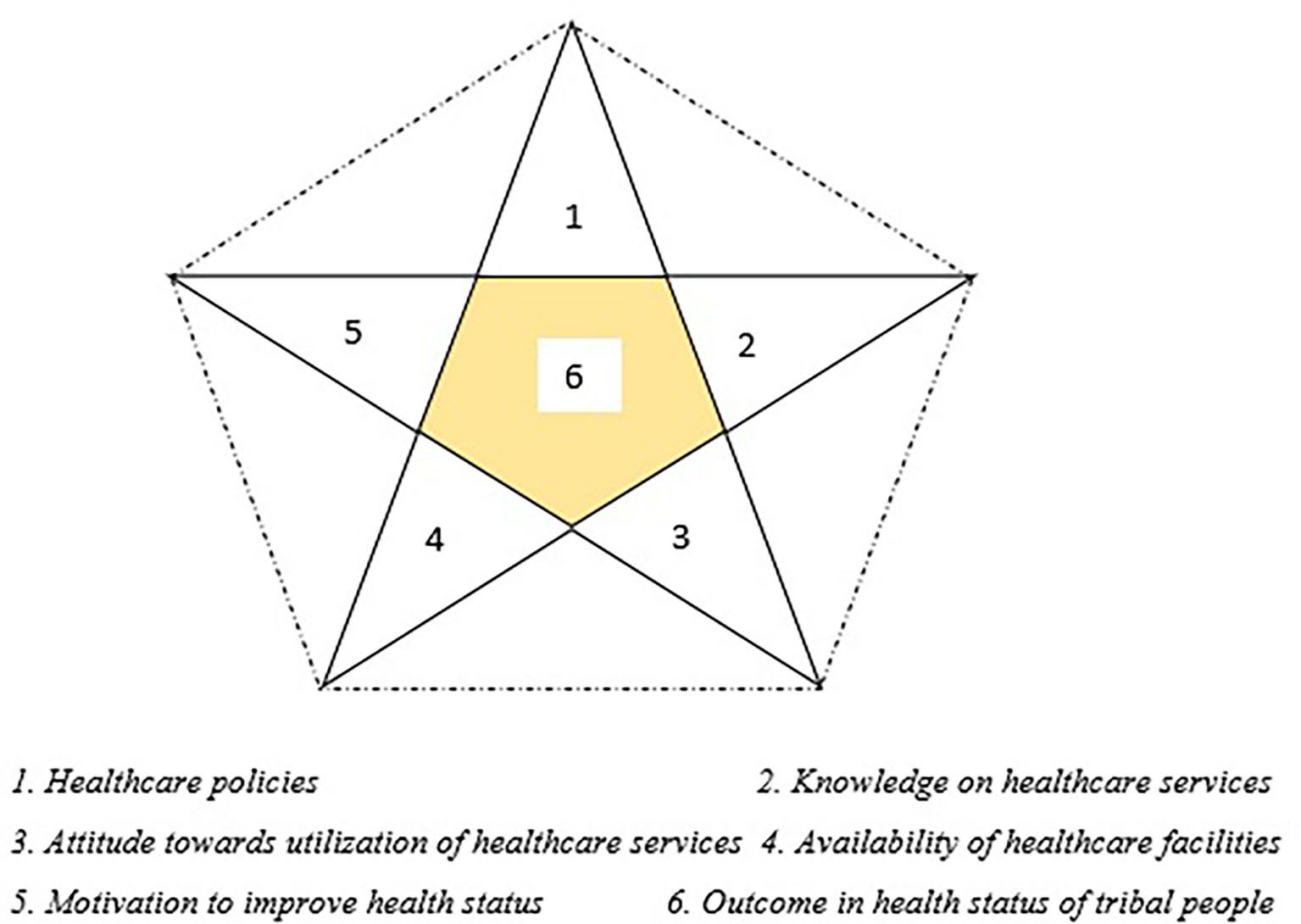
*“Star model of health status improvement”* to promote health status of tribal population. The “STAR Model of Health Status Improvement” for the tribal group is a conceptual framework, developed by the authors which focuses on, a comprehensive and multi-domain cooperative approach to enhance overall health status of the tribal community in Udupi district.

After the analysis of the qualitative descriptions of the research participants, we used the themes to incorporate an inductive approach in our qualitative inquiry. We identified five core areas that act as key components that surround the outcome of the tribal population’s health status. The inductive approach in this study enabled us to formulate a theory that explains the lacunae in the health status of the tribal population in the study area. We have adapted the interpretive inductionist approach, as this method enhances the balance between deductive and inductive approaches in qualitative studies. ^28^ The inductive theory in our research explains *“The outcome of health status of tribal population can be enhanced through a multidimensional approach”* which has been depicted through the ‘Star Model of Health Status Improvement to promote health status of the tribal population.’ ( Figure 3).

Various healthcare policies have made remarkable contributions to the modification of the existing healthcare practices and healthcare system approach towards tribal health. ^29^ Amendment of policies and the addition of new policies based on the requirements for tribal societal needs could certainly make a positive change towards a healthy lifestyle of them. ^30^ Knowledge of the tribal population hovers traditional healing and magical remediation. Ignorance and lack of awareness restrict them from using the available healthcare services and other schemes for them. On the other hand, the non-favouring attitude of the tribal population globally, which is supported by evidence, literally brings them back from the scientific and modern approach to treating illness and focusing on reducing maternal and child mortality rates. ^31^ Effective educational intervention programs by various agencies can change the attitude and knowledge of the tribal population towards a positive paradigm. However, the programs should be sustainable, and funding for such programs should be examined. Another crucial component in the “ *Star Model of Health Status Improvement”* is the availability of healthcare facilities in the tribal areas. As the majority of the tribal population dwells in the forest and hilly areas, it is difficult for the healthcare facilities to operate in those regions; hence, there are many hurdles to overcome to operate the facility in a smooth way. ^32^ Conversely, efficient planning and operational success with the supply of money, materials, and manpower by the government can meet this challenge. Another arm of the model points to the motivation of the tribal people. Even if the four elements mentioned above are adequate to meet the healthcare needs of tribal people, their motivation is most important to change their attitude and perception towards a healthy lifestyle by utilizing the facilities in the fullest way. ^33^ Awareness programs based on information education and communication (IEC) must help improve the health status of the tribal population. ^34^

Despite the existence of all these elements, the dotted lines represent the significance of the interrelation between these elements and their collaborative activities to help beneficiaries in the most effective way. ^35^ Any lack of communication or misrepresentation of information might lead to a deficiency in the effectiveness of healthcare-related programs; thus, it would weaken the other five elements, eventually reducing the motivation of tribal people and weakening their attitudes and practices. The central aspect of the model pertains to the health outcomes of the tribal population, which are significantly influenced by several essential factors represented by the five points of a star. Any modification to the current state of these critical components will undoubtedly affect the health status of tribal communities. Despite efforts made by governmental and private healthcare entities, certain community-based obstacles that diminish the efficacy of interventions aimed at these populations persist. Enhanced and purpose-driven programs that prioritize increasing awareness and fostering positive attitudes towards health improvement are likely to yield more effective results. ^36^

## Discussion

Access to quality healthcare is an essential requirement and fundamental right for every community. However, tribal populations face significant barriers to accessing healthcare services due to a variety of factors. Despite India’s significant advancements in technology and science in recent decades, its impact on tribal populations remains limited. Several studies conducted among tribal communities in India have emphasized the urgent need for a healthcare revolution and the importance of strengthening the implementation and evaluation of various programs and schemes aimed at improving the health of tribes. ^37, 38^ This research has provided new insights that have further enriched the existing knowledge in the field of tribal health.

This work aligns with ***Sustainable Development Goals (SDGs) 1,3 and 10,*** ^39^ and by focusing on these goals, contributes to creating a more equitable and inclusive healthcare system, ensuring that tribal communities receive the support and services they need. ^40^ This study highlights the issues of inequality and significance of culturally oriented healthcare, suggesting that implementation research could further explore these areas. It also emphasizes the importance of addressing the factors that influence health-seeking behavior in this PVTG.

The tribe in our research study, classified as a PVTG, receives various services from government initiatives and other agencies. Despite numerous initiatives and efforts, the current conditions have not succeeded in bringing about positive and sustainable changes in the tribal communities. This can be attributed to their strong adherence to deep rooted belief systems and traditions. Numerous studies have indicated that the healthcare services provided to such tribes are ineffective if they do not align with their traditional practices. Conversely, incorporating traditional healing practices into the local health system ensures better participation by indigenous communities. ^41– 43^ The present study revealed that tribal groups perceive the approach and services offered by stakeholders as lacking cultural orientation, leading to inefficient utilization. Consequently, this resulted in a decreased level of enthusiasm among the tribes in accepting the health care services. It is important to recognize that tribal populations worldwide adhere to unique health belief systems and traditions, making them resistant to the assimilation of external belief systems. ^43^ During the focus group discussions, some participants disclosed that for major illnesses and severe ailments, they engaged in religious rituals and traditional practices such as offering animal blood to their local deities. Tribal individuals’ health beliefs and practices are deeply ingrained in their customs and rituals. Therefore, bridging the gap between tribal communities and healthcare services can be achieved by providing culturally sensitive and accepted healthcare. The lack of education has been identified as a significant barrier to their appreciation and acceptance of healthcare services and other facilities. Despite the joint efforts of public and private initiatives, tribal groups often reject these advancements. Our findings agree with those of other researchers. ^41– 43^ Traditional healing practices valued by tribal populations, although accessible and affordable, are often underutilized and insufficiently integrated into modern medical systems. ^44^ Therefore, it is crucial to study their best health practices and explore the possibility of including them in modern medical approaches.

Tribal community representatives have voiced their concerns about the attitude of healthcare workers towards them during visits to healthcare facilities. Consequently, a gap has emerged between the tribal community and healthcare providers. Interestingly, several studies have emphasized that the closer the relationship between providers and beneficiaries, the greater the acceptance and benefits of the services by the people. ^41– 43^ In the current study, healthcare providers and stakeholders expressed concerns regarding the reluctance of tribal people to engage in health talks or programs, posing a significant challenge in delivering services to this population. Our study indicates that the success and benefits of these programmes and initiatives rely heavily on the active participation and involvement of the tribal community. The main reason behind this trend is the negative or unwelcoming attitude of tribal communities towards the healthcare system. However, this can be gradually improved through sustainable measures that enhance community involvement and participation. Some Western countries have already implemented such programs and witnessed positive outcomes. ^45^ It is crucial to integrate these programs with the SDGs to ensure long-term effectiveness. Stakeholders have also highlighted that despite the initiation of programs for tribal groups, the provision of necessary resources and manpower is often hindered by various socioeconomic factors. Researchers have emphasized the importance of meticulous planning and budget allocation to ensure the sustainability of community-oriented tribal health programs. ^45, 46^

### Limitation and contribution to future research

We conducted research in a PVTG in India, which has a declining population and many barriers in their access to healthcare services and schemes. As our study was conducted on a limited number of people, the generalizability of the studies has narrowed down. Although the other 74 PVTG in India face similar barriers and atrocities, each tribal group has its own culture and traditions, which should be considered in their particular context of social systems. This research is first of its kind among a PVTG in South India, and the findings of the study and the ‘Star Model of Health Status Improvement for tribal population’ which we have developed would indeed be an eye opener and helpful for further research among other tribal population in India or elsewhere globally.

## Conclusions

An in-depth qualitative inquiry among PVTG in South India explored the influence of social, economic, cultural, and contextual factors on healthcare experiences and behavior. The major obstacles to healthcare utilization include the lack of culturally sensitive care, discrimination within healthcare institutions, and limited collective advocacy capacity of the tribal community for services. The integration of study findings from both healthcare providers and the tribal community has also been instrumental in identifying key facilitators of the effective utilization of healthcare services. Effective administrative policies and well-coordinated activities can overcome these barriers and enhance the overall health and well-being of this declining tribal community. Moreover, actively involving them in the planning and implementation of these programs ensures cultural sensitivity, and significantly increases the likelihood of successful outcomes. Implementing healthcare programs for PVTG requires coordinated efforts from government and private partnerships, adequate planning, budgetary allocation, policy support, and administrative efficiency. Inter-sectoral cooperative activities, such as regular screening programs, vaccination programs, safe water, and sanitation awareness programs, would enable them to accept and use available resources to enhance their outlook and utilization of healthcare services. Furthermore, the study underscores the need for implementation research, evaluating the success and sustainability of such programs.

## Data protection issues

Because open posting of data on a repository was not included in the study information sheet at the time the survey was completed, data access will be granted once users have consented to the data-sharing agreement and provided written plans and justification for what was proposed with the data. As this qualitative study was conducted among a Particularly Vulnerable Tribal Group (PVTG), the data were not made available in the public domain to maintain confidentiality and data privacy. However, intermediary data can be available on request from the corresponding author, mentioning the reason for availing it.

## Reporting guidelines

The guidelines for reporting this qualitative study are available in Figshare: ISSM COREQ Checklist. Identifier. https://doi.org/10.6084/m9.figshare.29108882.v1

## Ethical statement: Ethical considerations

Permission was obtained from the Institutional Ethics Committee (KMC-KH IEC) of Kasturba Medical College, Manipal, Manipal Academy of Higher Education. (IEC No: 778/2019). A written informed consent was received from all participants. This research was conducted in accordance with the Declaration of Helsinki.

## Data Availability

Since the study was conducted among a PVTG, and their stakeholders, the authors have taken the precautions as per the guidelines by Declarations of Helsinki to protect the confidentiality and privacy of the participants and the data is provided based on that. The readers may contact the corresponding author for further information on this research on ranjitha.shetty@manipal.edu This project contains the following underlying data: Figshare: https://doi.org/10.6084/m9.figshare.29143349.v1
(a) Participant Information Sheet (PIS) and Informed Consent (IC) which were used to provide information to the participants about the purpose, procedures, benefits, risks, and their voluntary rights to participate/withdraw or withdraw from the study. (b) IDI and FGD guide and questionnaires. (c) Transcript of IDI and FGD. (d) COREQ checklist The data are available under the terms of the Creative Commons Attribution 4.0 International license (CC-BY 4.0).
