# Supplementary material for: Empowering Healthcare Access: Ethnographic Insights into Sustainable Solutions for a Particularly Vulnerable Tribal Group (PVTG) in South India- a Qualitative Research
Source: F1000Res. 2025 Jun 17;14:589. [Version 1] doi: 10.12688/f1000research.164492.1 (PMC12290442; doi:10.12688/f1000research.164492.1)
Supplement: Peer review history

# Reviewer report: Reviewer response for version 1

**DOI:** 10.5256/f1000research.181013.r392983

**Published:** 2025-07-24

**Authors:** Sudhir Prabhu H

**Affiliations:** 1 Department of Community Medicine, Father Muller Medical College, Mangalore, India

Researchers have tried to address a very important public health issue pertaining to healthcare accessibility for the tribal population with utmost scientific and ethical rigor.

 However there are few minor queries :

 1. How was the development of IDI and FGD guide or study tools for tribal study participants done ? Were local health or administrative team (serving the tribal area) part of this process? 

 2. What were the additional ethical safeguards taken in this study considering the study participants to be vulnerable ?

 3. Methods section under reflexivity and rigor on  trustworthiness of information provided mentions about "multiple data collection methods from stakeholders and beneficiaries" can research team confirm these methods used for triangulation.

 4. Did the tribals speak a different dialect and did the moderator and notekeeper during FGD or IDI understand or respond based on the needs for the same. Were issues pertaining to language a concern for the internal validity of the study ? if not how was this addressed?

Is the work clearly and accurately presented and does it cite the current literature?

Yes

If applicable, is the statistical analysis and its interpretation appropriate?

Yes

Are all the source data underlying the results available to ensure full reproducibility?

Yes

Is the study design appropriate and is the work technically sound?

Yes

Are the conclusions drawn adequately supported by the results?

Yes

Are sufficient details of methods and analysis provided to allow replication by others?

Yes

Reviewer Expertise:

Primary healthcare

I confirm that I have read this submission and believe that I have an appropriate level of expertise to confirm that it is of an acceptable scientific standard.

# Reviewer report: Reviewer response for version 1

**DOI:** 10.5256/f1000research.181013.r392985

**Published:** 2025-07-21

**Authors:** Dr. Harshal Pandve

**Affiliations:** 1 PCMC's Yashwantrao Chavan Memorial Hospital, Pune, India

It is very nicely done Ethnographic Research on Tribal Population. Methodology is clearly explained. Results are put in simple manner and are easy to understand. Discussion is adequate. Results are drawn from the results. Reference are mentioned as per standard protocol. Support and conflict of interest are mentioned. Author's contributions are clearly mentioned. I recommend to accept the article for indexing.

Is the work clearly and accurately presented and does it cite the current literature?

Yes

If applicable, is the statistical analysis and its interpretation appropriate?

Yes

Are all the source data underlying the results available to ensure full reproducibility?

Yes

Is the study design appropriate and is the work technically sound?

Yes

Are the conclusions drawn adequately supported by the results?

Yes

Are sufficient details of methods and analysis provided to allow replication by others?

Yes

Reviewer Expertise:

Community Medicine, Epidemiology, Qualitative Research

I confirm that I have read this submission and believe that I have an appropriate level of expertise to confirm that it is of an acceptable scientific standard.
